# Intention to imitate: Top-down effects on 4-year-olds’ neural processing of others’ actions^☆^

**DOI:** 10.1016/j.dcn.2020.100851

**Published:** 2020-08-27

**Authors:** Marlene Meyer, Hinke M. Endedijk, Sabine Hunnius

**Affiliations:** a Donders Institute for Brain, Cognition and Behaviour, Radboud University, the Netherlands; b Department of Psychology, University of Chicago, USA; c Education Science, Leiden University, Leiden, The Netherlands

**Keywords:** Top-down attention, Action observation, Young children, EEG, Motor activity, Imitation

## Abstract

From early in life, we activate our neural motor system when observing others’ actions. In adults, this so-called mirroring is modulated not only by the saliency of an action but also by top-down processes, like the intention to imitate it. Yet, it remains unknown whether neural processing of others’ actions can be modulated by top-down processes in young children who heavily rely on learning from observing and imitating others but also still develop top-down control skills. Using EEG, we examined whether the intention to imitate increases 4-year-olds’ motor activation while observing others’ actions. In a within-subjects design, children observed identical actions preceded by distinct instructions, namely to either imitate the action or to name the toy’s color. As motor activation index, children’s alpha (7−12 Hz) and beta (16−20 Hz) power over motor cortices was analyzed. The results revealed more motor activity reflected by significantly lower beta power for the Imitation compared to the Color-naming Task. The same conditional difference, although differently located, was detected for alpha power. Together, our results show that children’s neural processing of others’ actions was amplified by their intention to imitate the action. Thus, already at age 4 top-down attention to others’ actions can modulate neural action processing.

## Highlights

• Intention to imitate increases 4-year-olds’ neural motor activity during action observation.
• Top-down attention to others’ actions affects children’s neural action processing.
• We propose top-down effects are driven by an oscillation network with frontal theta predicting motor-related alpha/beta power.

## 1 Introduction

Paying attention to other people’s actions allows us to better understand, predict, and learn from what they do. Especially in early childhood, others’ actions provide rich information and form the basis for learning a diverse range of new skills. A substantial body of research in developmental psychology has contributed to our understanding of how children process others’ actions and how they imitate and learn from them (Hunnius and Bekkering, 2014; Marshall and Meltzoff, 2014; Meltzoff and Marshall, 2018). Given the plethora of actions happening around children every day, children face the challenge to focus on actions that allow them to extract useful information for their own behavior and to learn novel actions. Unravelling the neural underpinnings of how children process others’ actions, and in particular how to focus on action-related information, will inform ongoing investigations of action understanding and learning (Marshall and Meltzoff, 2014).

### 1.1 Neural underpinnings of processing others’ actions

Decades of cognitive neuroscience research have advanced our understanding of how actions are processed. Research on primates, human adults and children demonstrated that the neural activation found during observation of others’ actions closely resembles the neural patterns of performing the same action. Electrophysiological studies with adults and developmental populations, for instance, show that the alpha and beta rhythms overlaying sensorimotor regions are suppressed both during observation of another person’s actions as well as during action execution, with less power indicating more activation (e.g. Fox et al., 2016; Hari et al., 1998). This neural overlap, often called mirror mechanism or mirroring (Rizzolatti and Fogassi, 2014) is suggested to be the neural basis for understanding of and learning from others’ actions (Hunnius and Bekkering, 2014; Woodward and Gerson, 2014). Although the term ‘mirroring’ and different interpretations of this mechanism are under discussion (Csibra, 2008; Hickok, 2014) the finding itself is established based on more than a decade of research (e.g. Rizzolatti and Fogassi, 2014). The precise functionality of the mirror mechanism, however, is still matter of debate. In particular, the question whether the mirror mechanism is activated automatically during action observation or whether it is sensitive to top-down modulations like the relevance of actions to the observer has received attention recently (see Campbell and Cunnington, 2017 for a review). In contrast to bottom-up attention which relies on the properties of a stimulus, top-down attention to the environment is not driven by the features of a stimulus itself but rather by one’s prior experience, knowledge and internal goals (Katsuki and Constantinidis, 2014).

### 1.2 Top-down effects on processing others’ actions

Top-down processes like paying attention to actions relevant to one’s own behavior, for instance in order to reproduce an action or to coordinate with another person, amplify neural responses to the observed action. Studies with adults suggest that brain regions involved in action execution are activated more strongly during action perception when the observed action is relevant to the observer (Campbell and Cunnington, 2017). This evidence comes from studies in which adults watched actions after the explicit instruction to later reproduce the action rather than to passively view the same action or to perform a non-action related task (Grezes et al., 1998; Frey and Gerry, 2006; Muthukumaraswamy and Singh, 2008). For instance, the MEG study by Muthukumaraswamy and Singh (2008) found more motor activity as reflected by less beta power over motor cortices when adults observed an action in an imitation condition compared to a passive viewing condition. An EEG study with adults (Schuch et al., 2010) showed that observing a scene with action-related instructions led to increased motor activation as indexed by less sensorimotor alpha power compared to observing the same scene with instructions directed at color features. More indirectly, top-down effects on neural processing of others’ actions are observed in studies comparing different social contexts, for instance showing enhanced motor activity during action observation when adults were engaged in a social interaction with the observed person (Kilner et al., 2006; Kourtis et al., 2010; Ménoret et al., 2014).

### 1.3 Top-down effects on processing others’ actions in the developing brain?

Together these findings provide ample evidence for top-down modulations of action perception in adults and, specifically, for enhanced neural motor activity when actions are relevant to the observer. It is particularly in early childhood that observing others’ actions is valuable for learning and improving own skills. However, top-down control as reflected in cognitive flexibility and attentional control are still undergoing significant development in children’s first years of life (Rueda et al., 2004; Zelazo and Carlson, 2012). Thus, it is currently unknown whether neural processing of others’ actions in young children is modulated by top-down processes. Is young children’s neural motor activation increased for actions they intend to imitate? To our knowledge no previous developmental study has manipulated top-down processes on children’s neural processing of others’ actions employing manipulations in task demands like adult research has done (e.g. Frey and Gerry, 2006; Muthukumaraswamy and Singh, 2008). While relevant developmental EEG research has contributed to our understanding of how bottom-up differences in actions might affect children’s neural mirroring (Woodward and Gerson, 2014), little is known about top-down modulations. Recent findings suggest for instance that neural motor activation during action observation can differ depending on infants’ subsequent imitation responses (Filippi et al., 2016). This effect is likely reflecting bottom-up mechanisms, such that certain actions appear more salient to the infants thereby leading to both, enhanced neural processing and increased likelihood of subsequent imitation. Recent studies have also demonstrated that contextual information might effect infants’ neural motor activation during an anticipation phase (Southgate and Begus, 2013) and action observation phase (Langeloh et al., 2018; Patzwald et al., 2020). Furthermore, as in adult research (e.g. Kourtis et al., 2010), top-down effects on neural processing of others’ actions are observed more indirectly in a study comparing different social contexts. Meyer et al. (2011) demonstrated that 3-year-old children showed more motor activation (as indexed by less beta power) when observing an action partner perform an action than when observing the same action while not being engaged in the joint action. While these findings might suggest that young children’s neural motor system is sensitive to actions relevant for coordinating with others, it remains an indirect and thereby limited manipulation of top-down effects on processing others’ actions.

### 1.4 The current study

Here, we investigated the effect of top-down processes on children’s neural action processing. Based on previous adult work (Muthukumaraswamy and Singh, 2008; Schuch et al., 2010), we hypothesized that children’s neural motor activity during action observation would be increased when the action is relevant to their own actions (here: for imitating that action) compared to a control condition. In our within-subjects design, we manipulated top-down attention to an observed action by contrasting two action observation conditions in 4-year-old children. We tested children at the age of 4 because at that age children are able to follow explicit task instructions (pivotal for isolating top-down factors) but their attentional and cognitive control skills are still developing. Before watching a short video clip of an action, the 4-year-olds were asked to either subsequently imitate the action they saw (Imitation Task) or label the color of the toy that was acted on (Color-naming Task). The actions children observed in both conditions were identical. To assess children’s neural motor activation during action observation, we measured their EEG throughout the task. In our analysis, we then focused on the alpha (also called mu) and beta frequency bands since less power in these frequency bands over motor-related brain regions is associated with neural action processing (Fox et al., 2016; Hari et al., 1998).

## 2 Methods

### 2.1 Participants

Twenty-nine 4-year-old children (19 girls) with a mean age of 52 months (*SD* = 1.94 months) participated in this study. Three participants were excluded from the final sample due to an insufficient number of artifact-free trials (see *EEG data analysis* for details). Participants were recruited from a database with families who had indicated interest in participating in developmental studies living in the region of Nijmegen, a middle-sized city in the Netherlands. These participants belong to a subset of participants who took part in a longitudinal experiment (Endedijk et al., 2017). For none of the children atypical development was reported. Children were accompanied by their caregiver to the testing session. At the testing session, informed consent was obtained from the child’s legal guardian. After participation, children received a gift (book or 20 euros). This study was approved by the local ethics committee of Radboud University Nijmegen and consent was obtained according to the Declaration of Helsinki (1991; p. 1194).

### 2.2 Procedure

**Fig. 1:**
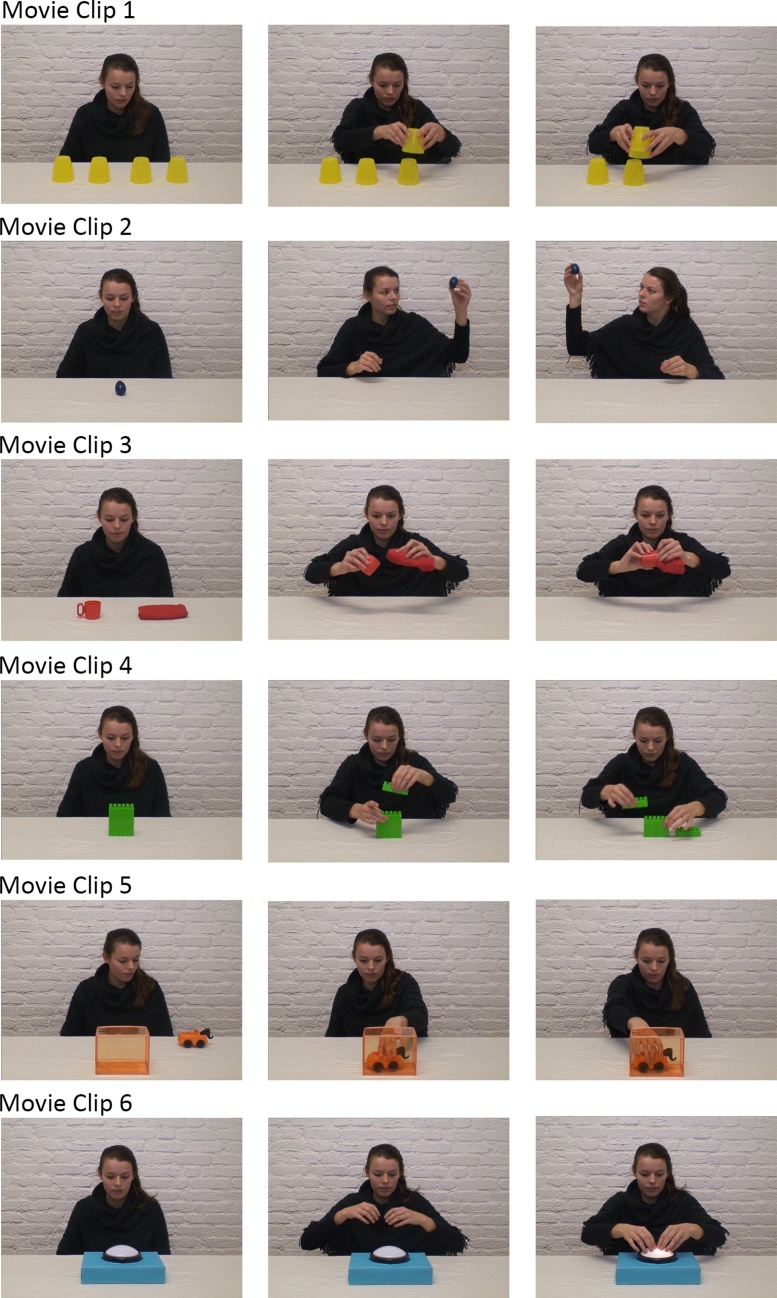
Illustration of all six actions demonstrated in the movie clips. The pictures represent snapshots of the movie clips with the first picture in each row showing the start frame and the following two pictures showing snapshots of the subsequent action unfolding.

Children and their caregivers were invited to an EEG testing session of approximately an hour. Upon arrival, caregivers and children were informed about the procedure and written consent was obtained from the child’s legal guardian. Then the child was fitted with a child-sized EEG cap containing 32 active electrodes (actiCap) arranged in a standard 10–20 system layout. The online reference was placed at electrode position FCz. For the EEG recording we used a BrainAmp DC EEG amplifier to digitize the signal at 500 Hz and band-pass filter the signal between 0.1 and 125 Hz. To reduce environmental noise on the EEG signal, parent and child were accompanied to a shielded room for the experimental part of the session. In the shielded room, children sat at a table with about 60 cm distance to a screen. The experimenter sat next to them so as to provide prompt instructions and parents observed the session from a chair in the back of the room. Children were presented with short movie clips (∼7−8 s). Action movie clips displayed a person performing one of six unique goal-directed actions using a toy. These actions were 1) stacking four upside-down cups to a tower, 2) shaking a rattle, 3) wiping the inside of a cup with a towel, 4) disassembling a stack of blocks, 5) moving a toy car into a box and out again and 6) turn on a lamp with two hands (see Fig. 1 for an illustration). Children watched the same action movie clips in two within-subjects conditions implemented by two distinct task instructions (Imitation Task, Color-naming Task). The two task instructions were presented in blocks, counter-balanced across participants. Before watching, in one block children were instructed to imitate the action they observed (Imitation Task) and in another block to later label the color of the toy that was acted on (Color-naming Task). Then children saw the action clip three times, each preceded by a fixation cross (1 s) before they got to execute the action (Imitation Task) or label the toy’s color (Color-Naming). Consistent with Endedijk et al. (2017) the fixation cross was used as baseline. As highlighted in Cuevas et al., 2014, it is favorable to have a baseline temporally close to the test events rather than using a baseline in a separate block. Children thus saw each unique action three times per condition, six times in total. Throughout the experimental session EEG was recorded to assess top-down effects on children’s neural response to action observation and to use their neural response during action execution to identify sample-specific frequency bands associated with action processing. Moreover, we video recorded children throughout the EEG recording for offline coding of children’s task performance and movements during action observation. Recordings from this experiment were also used as part of a longitudinal study on peer cooperation and neural mirroring (Endedijk et al., 2017) and to investigate the role of theta oscillations in task processing (Meyer et al., 2019). During the same recording session in a separate block the children also watched abstract movement clips (i.e. short screensaver clips) without any task. The abstract movement clips were played after every two unique actions. This was the same for both conditions. This was subject to a comparison in the study by Endedijk et al. (2017). For transparency, the findings for these stimuli can be found in Endedijk et al. (2017).

### 2.3 Behavioral analysis

Children’s behavior was coded offline to determine their task performance in the Imitation and Color-naming task (also see Endedijk et al., 2017). To score children’s imitation performance, each action was divided into three parts. The three parts were specified as follows: Movie Clip 1 – stack all cups, stack from left to right (or vice versa), pick up cups using both hands (at least 3 out of 4 times); Movie Clip 2 – shake the rattle, hold it up to the left side of the head, hold it up to the right side of the head; Movie Clip 3 – pick up cup, pick up towel, make sweeping movement with towel inside cup; Movie Clip 4 – take apart all blocks, alternately place blocks left and right (at least 3 times), arrange blocks in one line; Movie Clip 5 – move toy car into the box with one hand, change hands, move toy car out of box with other hand; Movie Clip 6 – activate light, use two hands to touch lamp, push the lamp twice. For each part of an action children imitated correctly, they received one point. Thus, for each action children could score a maximum of three points. To evaluate children’s performance in the Color-naming Task, each correct label of the toy color was scored with one point.

### 2.4 EEG data analysis

**Fig. 2:**
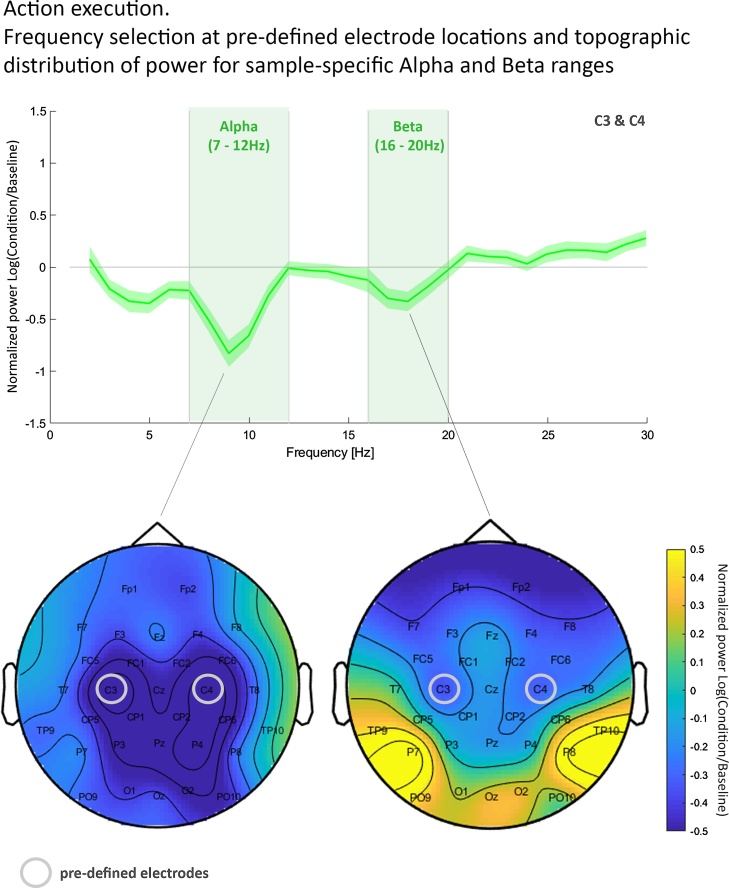
*Top row:* Normalized power at electrode sites C3 and C4 (averaged) shown as a function of frequency (Hz). Shaded areas around the mean difference line illustrate the standard error. The left green shaded area indicating the sample-specific frequency range identified for alpha (7–12 Hz), and the right green shaded area indicating the frequency range for beta (16–20 Hz). Negative normalized power values represent suppression during action execution with respect to baseline. *Bottom row:* Topographic distribution of the negative peak of normalized alpha power at 9 Hz (left) and beta power at 18 Hz (right) overlaid with circles visualizing the a priori defined electrode locations for sample-specific frequency selection and analyses (For interpretation of the references to colour in this figure legend, the reader is referred to the web version of this article.).

In accordance with previous studies, we focused in our analysis on two specific frequency bands: the alpha and beta frequency bands (Fox et al., 2016; Marshall and Meltzoff, 2011; Meyer et al., 2011, 2016). While power changes in both frequency bands are associated with processing of own and others’ actions, their functional role may differ. For instance, while alpha activity was proposed to link sensory and motor processes (Pineda, 2005), beta activity was suggested to be involved in monitoring and updating (Engel and Fries, 2010) as well as top-down predictive signaling (van Pelt et al., 2016). As their precise similarity and distinction remains underspecified, we examine these two frequency bands separately. We used the open source Matlab toolbox FieldTrip (Oostenveld et al., 2011) to conduct EEG data processing in line with Endedijk et al. (2017). More specifically, we first segmented the data in 1 s segments, separate for fixation cross (with 18 possible segments in total per condition) and action movie clip periods (with 144 possible segments in total per condition), eight segments per action movie clip. We then re-referenced the data to the average of all electrodes. Subsequently, all 1 s segments, experimental and baseline segments, in which children moved or looked away from the stimulus were discarded. Moving was defined as any fine or gross motor movement children performed during each 1 s segment. The remaining segments were visually inspected and EEG artifacts (e.g. due to noisy channels or eye blinks) were rejected. For three children no baseline trials for the Color-naming condition remained. Therefore, they were removed from the final analysis. For the remaining sample of 26 children, per child 65 segments remained on average for action observation in the Imitation condition (*SD* = 30), 59 segments for action observation in the Color-naming condition (*SD* = 33), with a minimum of 13 segments for the Imitation condition and a minimum of 20 segments for the Color-naming condition. On average, 7 segments for the baseline preceding action observation in the Imitation condition (*SD* = 3), and 6 segments for the baseline preceding action observation in the Color-naming condition (*SD* = 4) remained. Comparing the number of segments for experimental and baseline periods between conditions did not yield any evidence for a difference between conditions (Experimental: *t*(25) = 1.022, *p* = .316, *d* = .20; Baseline: *t*(25) = -1.113, *p* = .276, *d* = -.21). We used a DFT filter to remove line noise from the data, and we subtracted the mean signal of each segment from each time point of the segment to take out potential offset differences. To estimate spectral power we used Fast Fourier transform with a Hanning taper and finally calculated the average power for each child over all segments separately for each condition and corresponding baseline. As suggested by Cuevas et al. (2014) the neural signal recorded during action observation was normalized using a baseline which did not contain any action (here: fixation cross period). More specifically, we calculated the ratio of average power during action observation for each condition relative to the average baseline of that condition. Taking into account that ratios are not normally distributed, we then applied a log transform to the ratio data. This resulted in normalized values for all frequency bands. We focused on alpha and beta frequency ranges of electrodes overlaying sensorimotor regions (C3, C4) in line with previous adult and child research (Pfurtscheller and Da Silva, 1999; Endedijk et al., 2017). Note that analysis of alpha power was also part of supplementary analyses in Meyer et al. (2019). However, this article examined a different research question and the analyses involved different EEG processing steps (e.g. no baseline-correction). As frequency bands shift throughout early development, we used normalized power values from the action execution period to identify our sample-specific alpha and beta frequency ranges associated with action processing (see Fig. 2). For this purpose, we inspected normalized power values of the grand mean, averaged over central electrodes C3 and C4 between 3 Hz and 30 Hz. By determining frequency ranges with less power during action execution compared to baseline we identified the sample-specific frequency band of alpha at 7−12 Hz and of beta at 16−20 Hz (see Fig. 2). To compare children’s neural motor activity between the two action observation conditions (Imitation Task vs. Color-naming Task), we then extracted the normalized power values for these sample-specific bands averaged across central electrodes (C3/C4) for each condition. We hypothesized to find less alpha and beta power for the Imitation Task compared to the Color-naming Task. In our main, hypothesis-driven part of the analysis, we statistically compared the normalized power values between conditions using a paired samples *t*-test per frequency band. To further examine the topographic specificity of the effects we provide topographic plots of normalized power in the alpha and beta frequency range. Additionally, in a data-driven part of the analysis we then conducted a cluster-based permutation test per frequency band across all electrodes, to further explore differences between conditions across all sites. Comparable studies with children of the same age are rare and thus details such as electrode sites of the planned analyses were mostly based on either infant or adult literature.

## 3 Results

### 3.1 Behavioral results

In the Imitation Task, all children performed all actions. On average, children had an imitation score of 2.59 (range 1–3) out of a maximum of 3. In the Color-naming Task, one child labelled 3 out of 6, two children labelled 5 out of 6, and all remaining children labeled all colors correctly. Together, children performed at ceiling level on both tasks.

### 3.2 EEG results

**Fig. 3:**
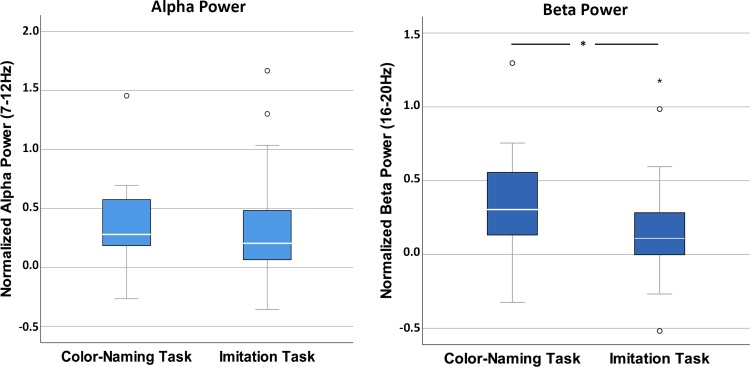
Boxplots displaying normalized alpha power (left) and normalized beta power (right) averaged over a priori defined electrodes C3 and C4 dependent on the within-subjects condition. Negative values reflect less power and positive more power with respect to baseline. The asterisk indicates a significant difference between the Color-naming and Imitation Task.

Fig. 3 illustrates normalized power in alpha (left) and beta (right) frequency bands for the two conditions, Imitation and Color-naming. Besides highlighting condition differences, the figure shows that across conditions there is no indication for power suppression (i.e. represented in negative values) in the alpha or beta band with respect to baseline. One-sample *t*-tests against zero rather suggest an increase in power with respect to baseline (alpha: Imitation condition, *t*(25) = 3.246, *p* < .05, *d* = .63; Color-naming condition, *t*(25) = 5.471, *p* < .05, *d* = 1.07; beta: Imitation condition, *t*(25) = 2.364, *p* < .05, *d* = .46; Color-naming condition, *t*(25) = 5.215, *p* < .05, *d* = 1.02). For more information on the neural response to action observation (averaged across conditions), a topographic plot and spectral distribution across the C3/C4 channels is provided in the Supplementary Material (Supplementary Fig. S1). Although the lack of overall suppression is unexpected, it is in line with a number of previous developmental studies (e.g. Marshall et al., 2013; Ruysschaert et al., 2013). To exclude the possibility that differences in the baseline are driving any effects, we ran a paired samples *t*-test for each frequency band comparing power values at C3/C4 across conditions in the baseline. Neither in the alpha (*t*(25) = -.103, *p* = .919, *d* = -.02), nor the beta range (*t*(25) = -.153, *p* = .138, *d* = -.03) did we find evidence for differences between the baselines.

#### 3.2.1 Alpha frequency band (7−12 Hz)

Based on our a priori hypothesis we tested for condition differences in children’s motor activity by comparing normalized power in the alpha range at central electrode sites (C3/C4). The paired samples *t*-test did not reveal any significant differences between the mean normalized power in alpha at electrode sites (C3/C4) between the Color-naming condition (*M* = .35, *SE* = .06) and the Imitation condition (*M* = .30, *SE* = .09), *t*(25) = .589, *p* = .561, *d* = .11. The results are illustrated in Fig. 3 (left). Thus, results of the hypothesis-driven analysis of the alpha frequency range at electrode sites (C3/C4) does not provide any evidence for increased motor activation during observation of an action with the intention to later imitate that action.

#### 3.2.2 Beta frequency band (16−20 Hz)

Analogously to alpha, we contrasted the two action observation conditions in the beta band. The paired samples *t*-test revealed a significant difference of the mean normalized power in beta at central electrode sites (C3/C4) between the Color-naming condition (*M* = .33, *SE* = .06) and the Imitation condition (*M* = .16, *SE* = .07), *t*(25) = 2.407, *p* = .024, *d* = .47. For an illustration of the results see Fig. 3 (right). These findings indicate significantly less beta power, thus stronger motor activation, when 4-year-olds watched another person’s actions with the intention to imitate that action compared to watching the same action with the intention to report visual aspects of the action (i.e. toy color).

#### 3.2.3 Topography of conditional differences

**Fig. 4:**
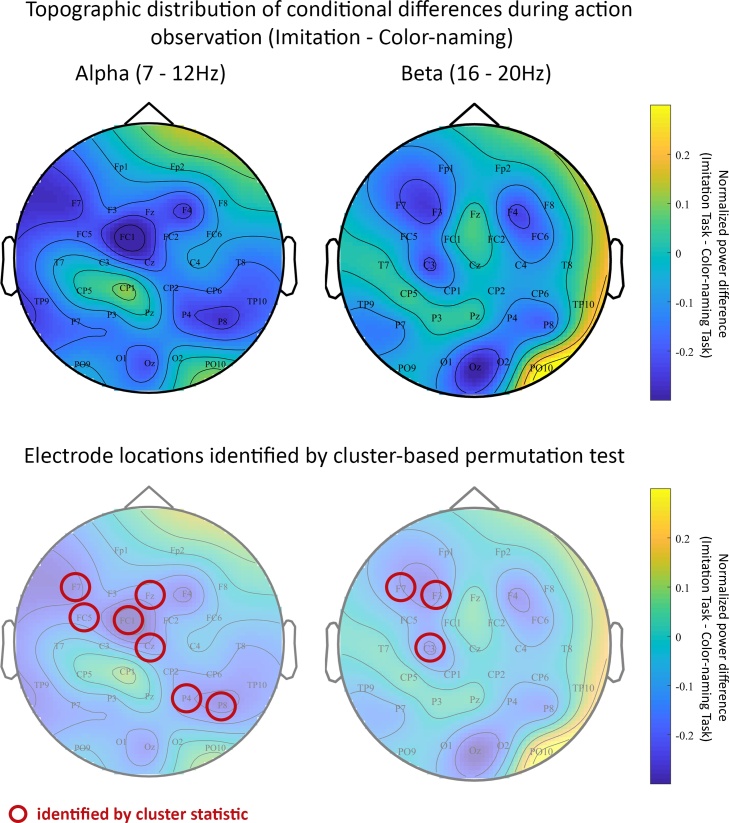
*Top row:* Topographic distribution of differences in normalized alpha power (left) and normalized beta power (right) between the Imitation condition and Color-naming condition. Cooler colors represent less power for the Imitation compared to the Color-naming condition. Warmer colors represent more power for the Imitation compared to the Color-naming condition. Less power in these frequency bands over sensorimotor regions is associated with more motor activity. *Bottom row:* Topographic distribution of conditional differences in normalized alpha power (left) and normalized beta power (right) overlaid with circles visualizing the electrode locations identified as part of a cluster based on the cluster-based permutation test.

Fig. 4 (top row) displays the topography of differences between the two action observation conditions (Imitation Task vs. Color-naming Task) in the alpha and beta frequency bands. As illustrated by cooler colors over left and right fronto-central sites, children’s beta power was less over the motor regions of the brain for the Imitation compared to the Color-naming task. This distribution of the effect is consistent with the a priori selected electrode sites (C3/C4). A similar data pattern is visible at lateral frontal and parietal-occipital sites.

Interestingly, also in the alpha frequency range normalized power appears to be less for the Imitation compared to the Color-naming task over motor regions. While the a priori defined sites of interest (C3/C4) did not reveal evidence for any conditional differences, the topographic distribution of this alpha contrast suggests an effect in the same hypothesized direction at more centrally located sites as a priori assumed. To examine condition differences across all electrode sites more rigorously, we conducted a data-driven cluster-based permutation test (Maris and Oostenveld, 2007) for both frequency ranges.

#### 3.2.4 Data-driven comparison of conditional differences across all electrodes

We explored conditional differences across all electrode sites by means of a cluster-based permutation test. As a result, the contrast for alpha reveals a negative cluster over fronto-central (F7, Fz, FC5, FC1, Cz) and right parietal (P4, P8) electrode sites. At this cluster, the difference between conditions was significant (*p* < .05). While we found no evidence for a difference in the a priori defined electrodes C3/C4, the negative cluster over slightly more frontal and midline central electrodes suggests that also alpha range activity was modulated by task instructions. Consistent with our beta findings from the hypothesis-driven analysis, these alpha findings indicate stronger neural motor activation when children watched an action with the intention to imitate it rather than to perform a non-action related task. In contrast to the beta effect, the alpha effect was located more along the midline in central regions rather than over left and right sensorimotor cortices. Also, there were no occipital clusters with a significant difference between conditions for alpha. Comparing beta power between conditions in a data-driven manner also yielded a negative cluster. Overlapping with the a priori defined electrodes (C3/C4) the cluster that was detected spreads over left fronto-central sites (F7, F3, C3). This cluster-based statistic comparing the conditions was marginally significant (*p* < .1). Thus, for beta, hypothesis-driven and data-driven results converge. Fig. 4 (bottom row) illustrates the distribution of all electrode locations identified by the cluster statistics. Additionally, Supplementary Fig. S2 displays the conditional difference across all frequencies (3−30 Hz) for the electrode clusters identified for the alpha and beta frequency band.

#### 3.2.5 Exploration of spectral distribution and topography of the negative peak in beta

**Fig. 5:**
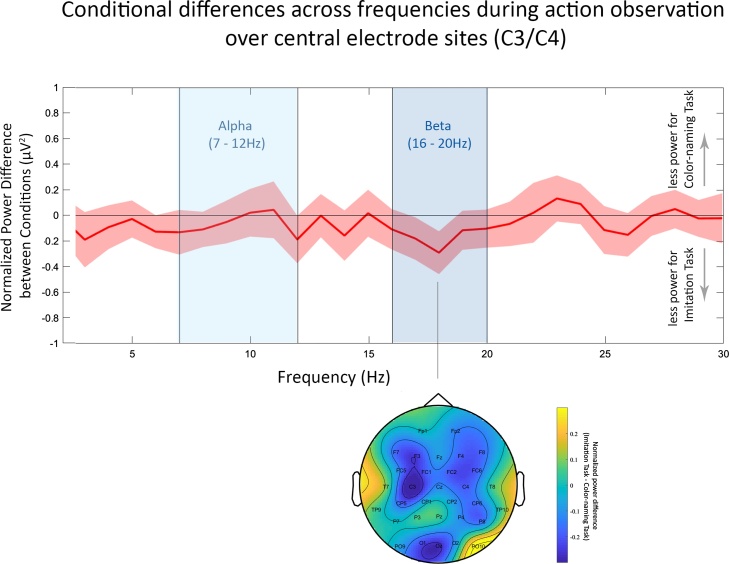
*Top row:* Normalized power difference between conditions at electrode sites C3 and C4 (averaged) shown as a function of frequency (Hz). Positive values represent less power for the Color-naming Task and negative values represent less power for the Imitation Task. Shaded areas around the mean difference line illustrate the standard error. The light blue shaded areas indicates the sample-specific frequency range for alpha (7-12 Hz), and the dark blue shaded area indicates the beta frequency range (16-20 Hz). *Bottom row:* Topographic distribution of the negative peak of the normalized difference in beta power identified at 18 Hz (right) (For interpretation of the references to colour in this figure legend, the reader is referred to the web version of this article.).

Besides the data-driven comparison, we further provide a depiction of the spectral specificity of the conditional difference across frequencies 3–30 Hz at the pre-defined electrode sites (C3/C4) and the topography of the negative peak in the beta power at 18 Hz in Fig. 5. Note that this topographic map of the beta effect is only for descriptive purposes and no statistical tests were conducted to avoid multiple testing of a previously identified effect. To further explore potential influences of the baseline, we provide the same illustration as direct contrast without baseline-correction between the two conditions in the Supplementary Material (Supplementary Fig. S3). The spectral and topographic distributions show the same pattern as our main effect, i.e. less power for the Imitation compared to the Color-naming Task in the beta frequency range with a peak distributed over fronto-central brain regions. This demonstrates the robustness of the condition difference we observe.

## 4 Discussion

In this study, we investigated whether 4-year-olds’ neural motor activation when observing others’ actions is modulated by top-down processes. Based on cognitive neuroscience research with adults (Muthukumaraswamy and Singh, 2008; Schuch et al., 2010), we hypothesized that children’s neural motor activity would be increased when watching an action with the intention to imitate it (Imitation Task) compared to when watching the same action with a non-action related task (Color-naming Task). Our planned and data-driven analyses provide converging evidence that in contrast to the Color-naming condition, children’s neural motor activation was increased when observing an action with the intention to imitate it. Our findings suggest that children processed the same action with more engagement of their neural motor system when it was relevant for their own subsequent behavior.

### 4.1 Intention to imitate increases 4-year-olds’ neural motor activation

We analyzed the power in two frequency bands, alpha and beta, which are well-established indices of neural motor activation (e.g. Fox et al., 2016; Hari et al., 1998; Pfurtscheller and Da Silva, 1999). Our planned analyses of these frequency bands over left and right sensorimotor cortices revealed more motor activity indexed by significantly lower baseline-corrected beta power for the Imitation Task compared to the Color-naming Task. The topography of the negative peak of this beta effect illustrates that the modulation is strongest at electrode sites overlaying motor cortices and spreads out to lateral frontal sites overlaying premotor regions as well as parietal sites overlaying sensorimotor cortices. This suggests top-down effects in brain regions involved in action processing such as motor and premotor cortices. Moreover, this is consistent with previous findings of top-down effects on beta oscillations reflecting modulations of motor activity when adults observe an action with the intention to reproduce it (Muthukumaraswamy and Singh, 2008). It is further in line with beta effects induced by different social contexts in children and adults (Kourtis et al., 2010; Ménoret et al., 2014; Meyer et al., 2011). Although only marginally significant, outcomes of the data-driven analysis of beta power across all electrodes overlap and converge with results from the planned analyses. The frontal topography reported in the data-driven analysis also fits with the frontal topography during children’s action execution (see Fig. 2) and is in line with evidence of top-down connections of signals in the beta frequency range between prefrontal and parietal regions (van Pelt et al., 2016).

In contrast, there was no initial evidence for condition differences in the alpha frequency band. However, data-driven cluster-based tests revealed that rather than over left and right sensorimotor cortices (as used in the planned comparison) there were condition differences in the alpha band over central-midline areas, left frontal and right parietal sites. Like in the beta effect, baseline-corrected power in the alpha range (although at more midline-central sites) was less for the Imitation Task compared to the Color-naming Task. The topography of the alpha effect is comparable to that found by Schuch et al. (2010) when contrasting alpha range activity of adults solving action-related and color-related tasks. Moreover, the fronto-parietal results fit with the notion that fronto-parietal networks reflect action processing (Pineda, 2005). The topographic distribution of alpha and beta power, in particular with respect to their frontal activation sites might further suggest links between movement learning and planning with premotor cortices. In accordance with that, Suchan et al. (2008) found that adults’ premotor cortex is active when they were asked to observe actions to later imitate them (see also Frey and Gerry, 2006). Similarly, this fronto-central and partially parietal distribution also fits with other adult neuroimaging studies on action observation and observational learning (e.g. Buccino et al., 2001; Cross et al., 2009; Molenberghs et al., 2012). Besides this, the cluster-based tests did not provide any evidence for condition differences in occipital channels (i.e. no condition differences at occipital clusters reached significance) suggesting that sensorimotor alpha rather than occipital alpha was affected by children’s intention to imitate. All in all, the results provide evidence for top-down attentional effects on neural processing of others’ actions already in early childhood.

### 4.2 Limitations of the current study

Contrary to what was expected (see e.g., Fox et al., 2016), our study did not yield evidence for overall suppression with respect to baseline in alpha or beta frequency ranges (see Fig. 3). However, several other developmental EEG studies report similar results to ours (Köster et al., 2020; Marshall et al., 2013; Ruysschaert et al., 2013; also cf. Endedijk et al., 2017, for a detailed discussion). A potential explanation might be that observing televised compared to live actions elicits overall less motor activity (Shimada and Hiraki, 2006). Also, one might speculate that children actively inhibited their motor system because they were asked to sit still during the action videos, which might have resulted in an increase in power with respect to baseline. This fits with research showing an increase in sensorimotor alpha when adults are asked to inhibit an action, an effect stronger in younger compared to older adults (e.g. Bönstrup et al., 2015). Another possibility is that the task instruction which preceded the action observation might have led children to activate their motor system already during the baseline phase, which in turn might have led to a lack of suppression with respect to the action clips. This hypothesis, however, is not supported by our data (see Supplementary Material). Despite this unexpected outcome, all effects of conditional differences in our within-subjects contrast are in the hypothesized direction, with less power in the Imitation Task compared to the Color-naming Task. We are confident that irrespective of the overall motor activation with respect to baseline, the relative difference in motor activation between conditions, validly reflects differences in children’s neural processing of others’ actions. Another potential limitation of the current study is the loss of temporal information in the analysis. Since addressing the current research question did not require a time-resolved analysis, data of each stimulus movie were epoched in 1 s segments. This allowed us to include more data by not having to discard entire 7−8 second data epochs when only a small fraction was artifacted. While that allowed for more robust results, this analysis decision also limits the current findings because temporal information is lost. As we suggest below, future research could take this into account by using shorter movie clips such that temporal information is retained while keeping data loss at a minimum.

### 4.3 Top-down attention to action and imitation in development

The current findings not only add to adult literature but also extend the growing body of developmental literature on the role of mirroring for children’s imitation and learning from others (Hunnius and Bekkering, 2014; Marshall and Meltzoff, 2014; Meltzoff and Marshall, 2018; Woodward and Gerson, 2014). As summarized by Marshall and Meltzoff (2014), converging evidence suggests a tight link between imitating others’ actions and the neural processing of others’ actions from early in life. In line with this, recent results from an EEG study with 7-month-old infants show that neural motor activation to another person’s actions is stronger when it preceded infants’ subsequent imitation of the observed action (Filippi et al., 2016). This might be due to infants preferring one action over another (bottom-up influence) or it might result from infants’ prior intention to imitate what they are about to see (top-down influence). Important in the distinction between bottom-up and top-down attention is that bottom-up attention is solely caused by the properties of a given stimulus, such as its visual appearance (Katsuki and Constantinidis, 2014). While previous findings cannot dissociate between these alternative interpretations, the current results can exclude stimulus saliency as a driver of enhanced neural activation because the observed actions were identical across conditions. This allowed us to study the neural effect of having the prior intention to imitate an observed action in isolation. Since children’s performance in both tasks was at ceiling level, we could not assess potential beneficial effects of selective neural enhancement for imitation performance on an individual differences basis. Although beyond the scope of the current study, one might expect amplified neural motor activity to predict better imitation performance given prior work linking attentional effects reflected in alpha and beta oscillations and overt performance in adults (Haegens et al., 2011; van Ede et al., 2012) and recent infant EEG work (Filippi et al., 2016). Relatedly, in adults more precise imitation performance is related to alpha power suppression when later observing the previously imitated action again, further supporting a tight link between neural motor activity during action observation and imitation performance (Marshall et al., 2009). Together this might imply that instruction prior to demonstrating an action could not only enhance children’s neural response during action perception but also has the potential to affect children’s action learning. In infancy, at an age at which children cannot be instructed explicitly, using other forms of highlighting the relevance of the action and engaging the child might have similar effects on children’s neural processing of others’ actions. Strategies such as social engagement through gaze cues (Michel et al., 2015), turn-taking (Meyer et al., 2011) or infant-directed behaviors like infant-directed speech (Zhang et al., 2011) and infant-directed actions (van Schaik et al., 2019) might help to underline the relevance of an action for children too young to be explicitly instructed.

Although the focus of the current study was on the effect of top-down attention on the processing of others’ actions, when those were important for children, one might speculate on how top-down attention might have influenced children’s processing in the Color-Naming task. For instance, children might have paid particular attention to the color of the toy instead of the actions, leading to enhanced visual processing. Modulation of alpha power over occipital sites is thought to reflect enhanced visual attention to a stimulus (Herring et al., 2015). Therefore, one might have expected occipital alpha power to be suppressed in the Color-naming compared to Imitation condition. However, as apparent from the results of our data-driven comparison in the alpha range and Fig. 4, our data do not seem to support this speculation. For discussions on potential language-related effects on top-down processing in the theta frequency range see Meyer et al. (2019).

### 4.4 Top-down attention and the role of motor-related brain areas

Do top-down attentional effects in this context originate from and are they confined to motor-related brain regions such as (pre-)motor and sensorimotor areas or is the modulation we observe in the 4-year-olds’ neural motor activity a downstream result stemming from a more wide-spread attentional network? Although the current data cannot provide an ultimate answer to this question, previous theoretical and empirical work in cognitive neuroscience suggests a network of cortical oscillations spanning different brain regions involved in top-down processes. As proposed by Clayton et al. (2015) and Cavanagh and Frank (2014), supramodal theta oscillations from frontomedial brain regions might modulate modality-specific activity for instance in alpha and beta oscillations downstream. In line with this, van Ede et al. (2017) found top-down control effects in an MEG study in which adults had to either solve a visual or tactile working memory task. Their results show that medial prefrontal theta synchronization predicts subsequent modality-specific alpha and beta suppression (in visual and somatosensory regions, respectively). Consistent with this idea, results of post-hoc analyses of the current dataset showing that frontomedial theta power was modulated by task engagement and task demands (Meyer et al., 2019) hints at similar networks being at play in young children. Our design and limited number of trials did not allow us to conduct the same trial-wise correlational analysis as in van Ede et al. (2017) to systematically investigate the potential relation between theta synchronization in frontomedial sites and alpha and beta power over premotor and sensorimotor regions in the current data. Although optimal for addressing the main question of this study, the current presentation of three subsequent movie clips and the segmenting of each movie clip in 1 s segments resulted in the loss of time-locking critical for a trial-wise analysis as in van Ede et al. (2017). Moreover, van Ede et al. (2017) presented close to 500 trials to their adult participants which is far beyond the number of trials in the current study with 4-year-olds. While trial-level analyses were not possible with the current data we did conduct a first exploratory analysis on the subject-level. More specifically, we correlated frontal theta power (3−6 Hz) at Fz prior and during action observation (averaged across conditions) with the normalized alpha and beta power difference between conditions (Imitation – Color-naming Task) at C3/C4 (see Supplementary Analyses and Supplementary Figs. S4-S7). This exploratory analysis yielded a negative correlation between frontal theta and the central beta power effect, which provides a first indication that frontal theta power predicts subsequent top-down effects reflected in central beta oscillations. Rather than conclusive evidence, these exploratory results provide more leverage to pursuing a systematic investigation of this question. Future investigations are needed to test the idea that top-down effects on action processing involve a cortical oscillation network in which frontomedial theta synchronization predicts alpha/beta power effects in premotor and sensorimotor regions. For instance, by having very short movie clips (of about 2–3 seconds) each immediately followed by the response of the child, more time-locked data could be obtained. Moreover, testing a large number participants would further help to compensate for the lower number of trials inevitable when testing 4-year-old children. Addressing this type of oscillatory framework is particularly interesting in developmental populations given the drastic changes, functionally and structurally, of frontomedial brain regions such as medial frontal and anterior cingulate cortex which are thought to generate theta oscillations. This opens up an avenue with potential to inform questions from both, the field of developmental psychology and cognitive neuroscience. For instance, does the emergence of top-down attentional effects on action processing change as a function of structural development in frontal brain regions or rather motor cortical regions which mature significantly earlier in development? How does this predict children’s imitation behavior? Moreover, research on the precise functionality of oscillatory models of top-down attention might benefit from isolating the role of frontomedial theta and pre-motor and sensorimotor alpha/beta by harnessing the differences in developmental trajectories of frontal and sensorimotor cortices.

## 5 Conclusion

The current findings with 4-year-old children provide the first evidence that top-down attention to another person’s action modulates young children’s neural processing of that action. In particular, we found higher motor activation as indexed by alpha and beta rhythm activity when children observed an action with the intention to imitate it rather than solving a non-action related task (labeling the color of a toy). This suggests that already young children flexibly process others’ actions depending on the relevance of the observed actions for their own behavior.

## Author contributions

MM, HME and SH jointly developed the study concept and design. MM and HME collected the data. MM and HME performed data analyses and all authors interpreted the data. MM drafted the manuscript, and HME and SH contributed critical revisions. All authors approved the final version of the manuscript for submission.

## Declaration of Competing Interest

The authors report no declarations of interest.
