# Supplementary material for: Intention to imitate: Top-down effects on 4-year-olds’ neural processing of others’ actions
Source: Dev Cogn Neurosci. 2020 Aug 27;45:100851. doi: 10.1016/j.dcn.2020.100851 (PMC7481529; doi:10.1016/j.dcn.2020.100851)
Supplement: Supplementary file 1 [file mmc1.docx]

***Supplementary Material***

**Intention to imitate:**

**Top-down effects on 4-year-olds’ neural processing of others’ actions**

**Marlene Meyer^1,2^, Hinke M. Endedijk^3^, Sabine Hunnius^1^**

**^1^**Donders Institute for Brain, Cognition and Behaviour, Radboud University, The Netherlands

**^2^**Department of Psychology, University of Chicago, USA

**^3^**Education Science, Leiden University, Leiden, The Netherlands

***Supplementary analysis***

***Analysis baseline***

To explore the possibility the children’s motor activation was already higher during baseline periods when having a task, we compared the baseline (fixation cross) periods of the Imitation Task and Color-Naming Task with the fixation cross periods when children did not have a task, preceding the abstract movement clips. One would expect that if it holds that children already activate their motor system in anticipation of performing a task, there should be less power in the fixation cross period of the Imitation Task and potentially Color-naming Task compared to the No Task fixation cross period. For this purpose, we conducted a repeated measures ANOVA for alpha and beta power during the fixation cross periods at central electrodes (averaged C3/C4) with Condition (Imitation, Color-Naming, No task) as within-subjects factor. As a result, we found no indications for a significant difference in alpha (*F*(2,50) = 2.36, *p* > .05 η_p_^2^= .009) or in beta power (*F*(2,50) = 1.93, *p* > .05 η_p_^2^= .072). Alpha: Imitation Task, M = 7.6, SD = 5.3; Color-Naming Task, M = 7.5, SD = 5.4; No task, M = 7.0, SD = 5.0; Beta: Imitation Task, M = 1.07, SD = .70; Color-Naming Task, M = .96, SD = .59; No task, M = .90, SD = .54. Thus, there is no empirical support for this hypothesis.

***Correlational analysis of frontal theta and central alpha/beta effects***

Besides this, we ran an exploratory correlational analysis to examine whether frontal theta power (3-6 Hz) prior to and during action observation is related to the conditional difference in alpha and beta power overlaying motor areas. For this purpose, we extracted log-transformed theta power values (3-6Hz) for the pre-stimulus fixation cross period and the action observation period, averaged across conditions at site Fz. Theta power at this topographic location was previously shown to be modulated by top-down processing (van Ede, Jensen & Maris, 2017; Meyer et al., 2019). Theta values were then entered in Pearson correlations with normalized alpha and beta power difference values between conditions (Imitation – Color-naming Task) at C3/C4. The results are illustrated in Supplementary Figures S4-S7. For alpha, no relation with frontal theta is apparent (correlated with pre-stimulus theta: *r* = -.150, *p* > .05; action observation theta: *r* = -.159, *p* > .05). This is not surprising given that central alpha at pre-defined electrodes C3/C4 did also not show a condition difference. For beta, however, the overall pattern suggests a negative relation between frontal theta and central beta effects such that higher frontal theta power predicts a larger top-down effect on action processing as reflected by less beta power when children have to later imitate an action. This correlation is statistically significant when removing the outlier visible in Supplementary Figures S6 and S7 (correlated with pre-stimulus theta: *r* = -.436, *p* = .029; action observation theta: *r* = -.552, *p* = .004) but does not reach significance when including the outlier (correlated with pre-stimulus theta: *r* = -.202, *p* > .05; action observation theta: *r* = -.223, *p* > .05). With caution, one might interpret this as first indication that a network including frontal theta and central beta plays a role for top-down attention in action processing in young children.

***Supplementary figures***


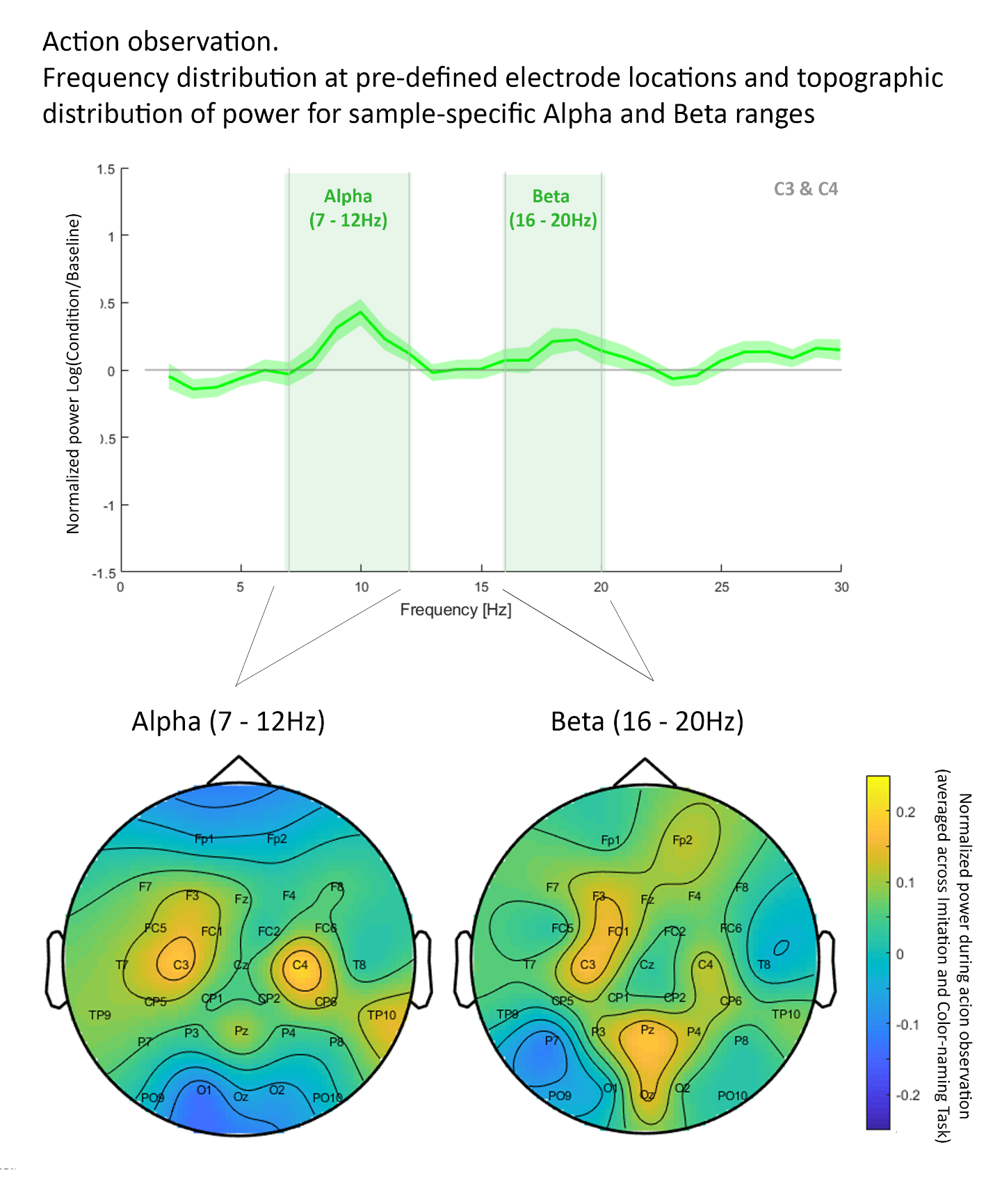


***Supplementary Figure S1.*** *Top row:* Normalized power at electrode sites C3 and C4 (averaged) shown as a function of frequency (Hz). Shaded areas around the mean difference line illustrate the standard error. The left green shaded area indicating the sample-specific frequency range identified for alpha (7–12 Hz), and the right green shaded area indicating the frequency range for beta (16–20 Hz). Negative normalized power values represent suppression during action observation (averaged across Imitation and Color-Naming conditions) with respect to baseline. *Bottom row:* Topographic distribution of normalized alpha power at 7-12Hz (left) and beta power at 16-20Hz (right).

**
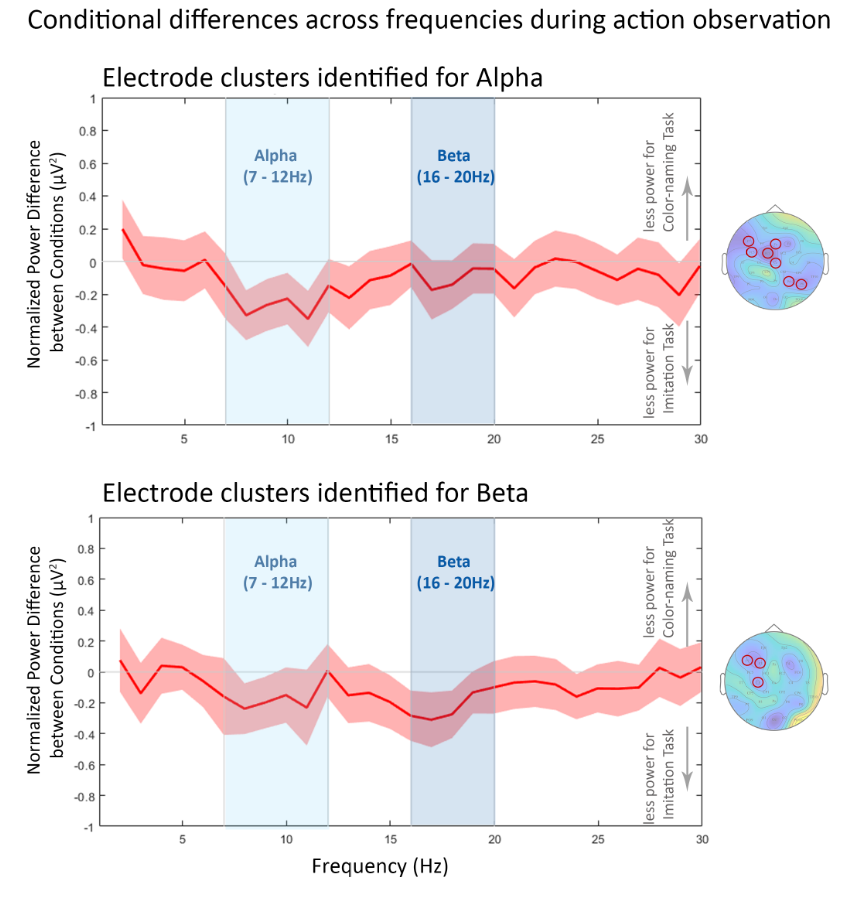
**

***Supplementary Figure S2.*** *Top row:* Normalized power difference between conditions at electrode clusters identified for the alpha range shown as a function of frequency (Hz). Identified clusters are illustrated in the topography map to the right of the panel. Positive values represent less power for the Color-naming Task and negative values represent less power for the Imitation Task. Shaded areas around the mean difference line illustrate the standard error. The light blue shaded areas indicates the sample-specific frequency range for alpha (7-12 Hz), and the dark blue shaded area indicates the beta frequency range (16-20 Hz). *Bottom row:* Normalized power difference between conditions at electrode clusters identified for the beta range shown as a function of frequency (Hz). Identified clusters are illustrated in the topography map to the right of the panel. Positive values represent less power for the Color-naming Task and negative values represent less power for the Imitation Task. Shaded areas around the mean difference line illustrate the standard error. The light blue shaded areas indicates the sample-specific frequency range for alpha (7-12 Hz), and the dark blue shaded area indicates the beta frequency range (16-20 Hz).


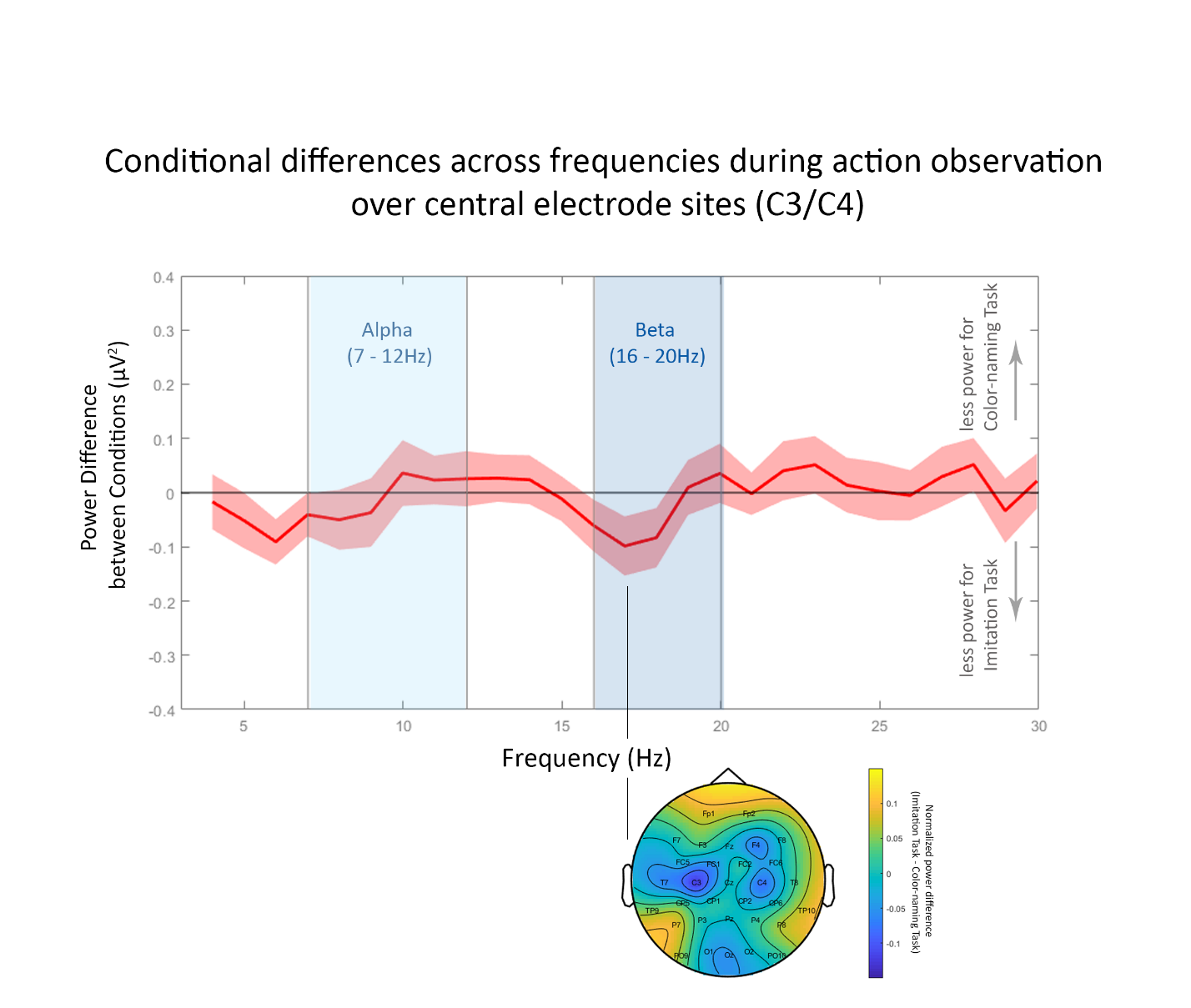


***Supplementary Figure S3.*** *Top row:* Power difference (not baseline-corrected) between conditions at electrode sites C3 and C4 (averaged) shown as a function of frequency (Hz). Positive values represent less power for the Color-naming Task and negative values represent less power for the Imitation Task. Shaded areas around the mean difference line illustrate the standard error. The light blue shaded areas indicates the sample-specific frequency range for alpha (7-12 Hz), and the dark blue shaded area indicates the beta frequency range (16-20 Hz). *Bottom row:* Topographic distribution of the negative peak of the difference in beta power identified at 17Hz (right).


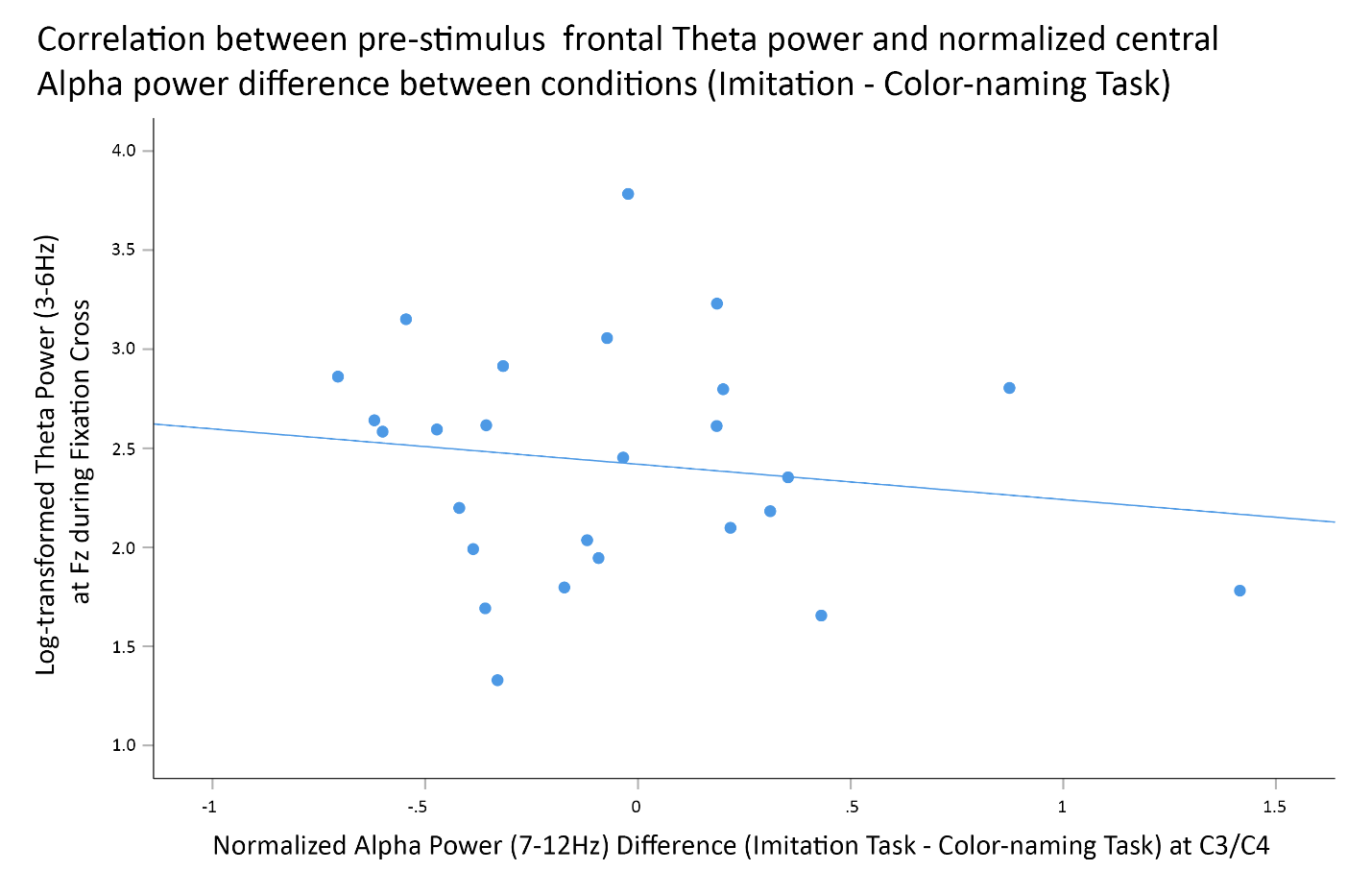


***Supplementary Figure S4.*** Scatterplot illustrating the relation between normalized alpha power (7–12 Hz) difference between Imitation and Color-naming Task at C3 and C4 (averaged) and pre-stimulus theta power (3-6Hz) at Fz. Dots represent individual participants.

***
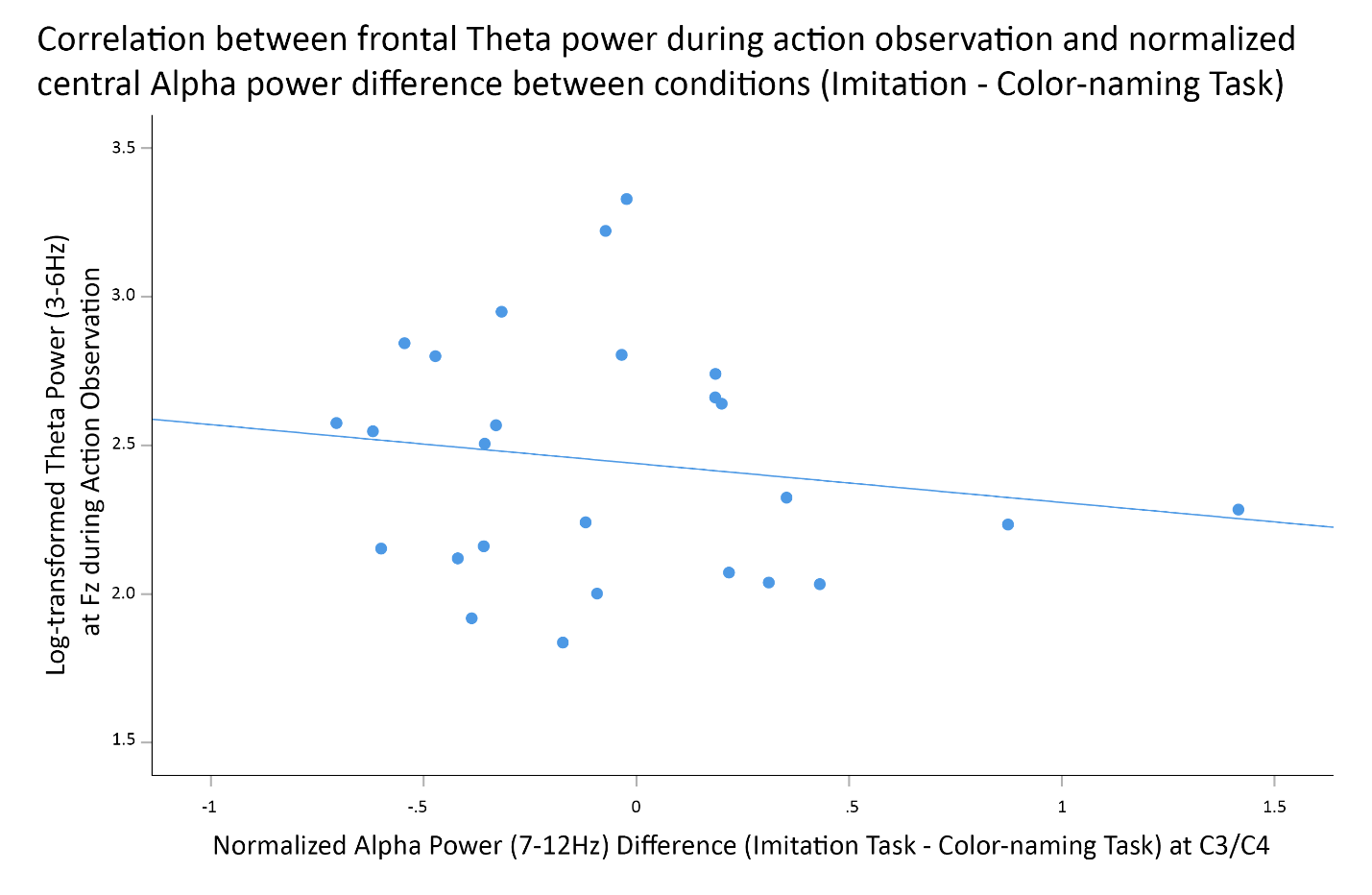
Supplementary Figure S5.*** Scatterplot illustrating the relation between normalized alpha power (7–12 Hz) difference between Imitation and Color-naming Task at C3 and C4 (averaged) and theta power (3-6Hz) at Fz during action observation (averaged across conditions). Dots represent individual participants.

***
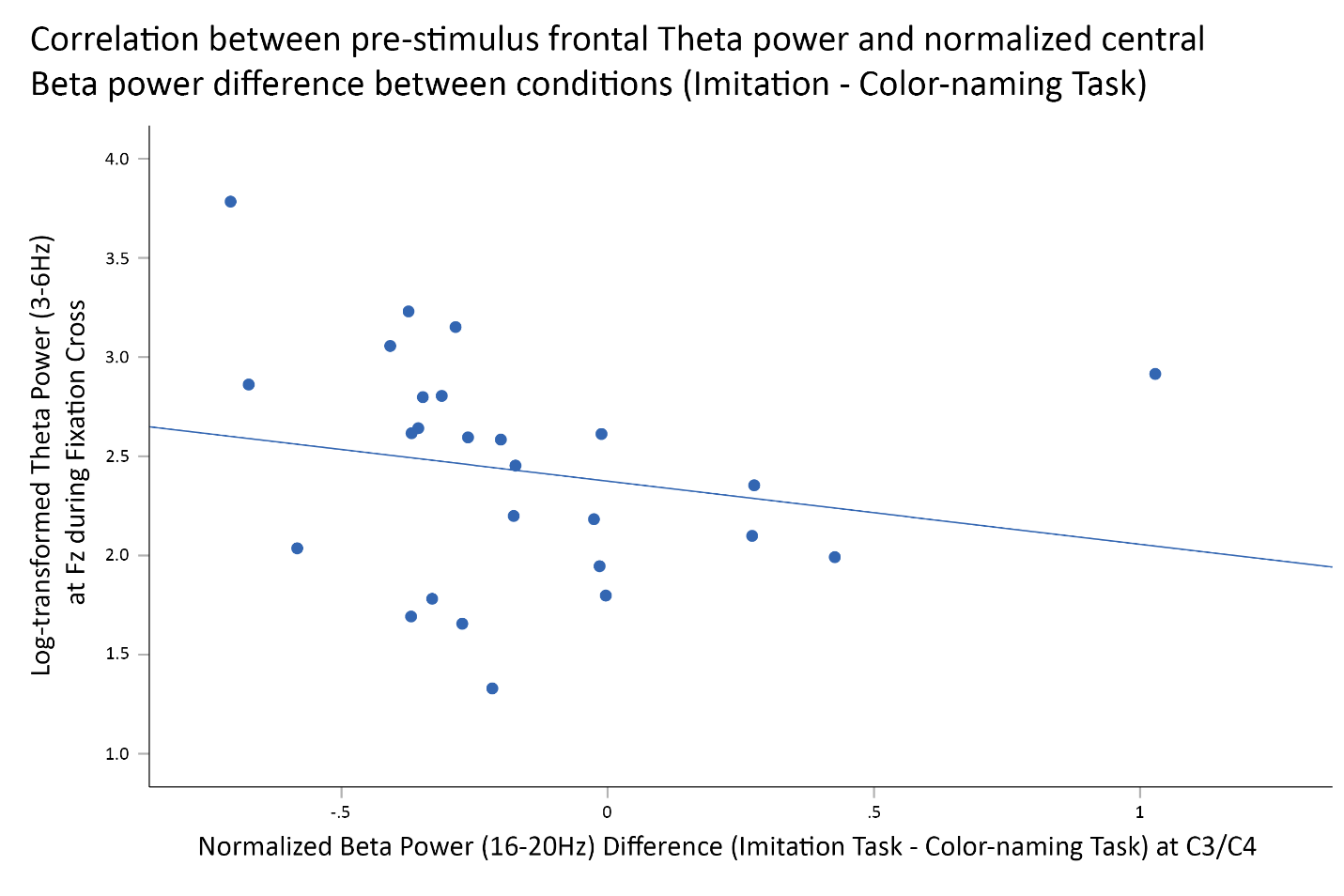
Supplementary Figure S6.*** Scatterplot illustrating the relation between normalized beta power (16-20Hz) difference between Imitation and Color-naming Task at C3 and C4 (averaged) and pre-stimulus theta power (3-6Hz) at Fz. Dots represent individual participants.

***
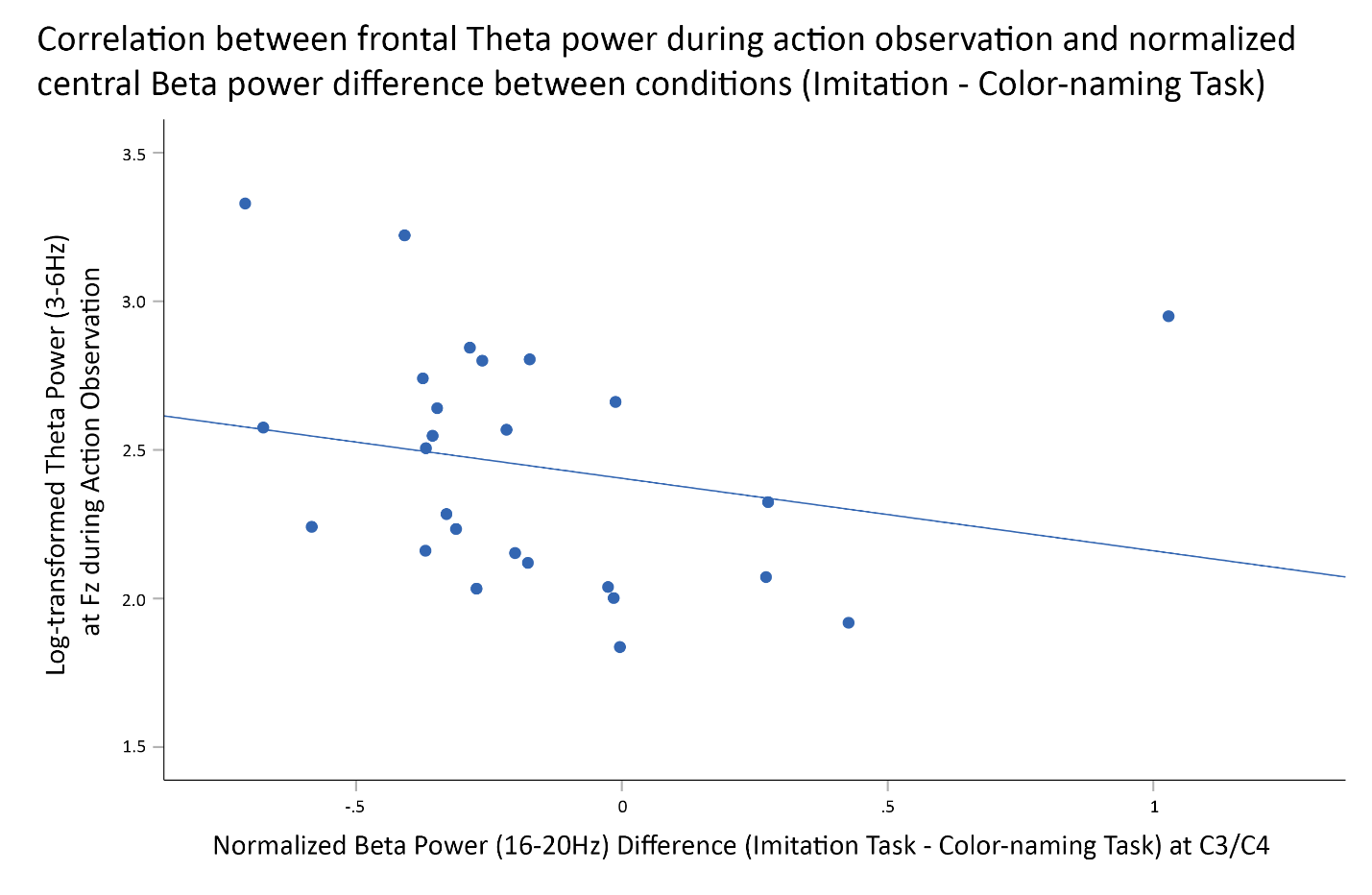
Supplementary Figure S7.*** Scatterplot illustrating the relation between normalized beta power (16-20Hz) difference between Imitation and Color-naming Task at C3 and C4 (averaged) and theta power (3-6Hz) at Fz during action observation (averaged across conditions). Dots represent individual participants.
